# Untangling the Emotional Intelligence-Suicidal Ideation Connection: The Role of Cognitive Emotion Regulation Strategies in Adolescents

**DOI:** 10.3390/jcm9103116

**Published:** 2020-09-26

**Authors:** Cirenia Quintana-Orts, Sergio Mérida-López, Lourdes Rey, Félix Neto, Natalio Extremera

**Affiliations:** 1 Department of Personality, Evaluation and Psychological Treatment, University of Granada, Campus Ceuta, Cortadura del Valle s/n, 51001 Ceuta, Spain; 2 Department of Social Psychology, Social Work, Social Anthropology and East Asian Studies, University of Malaga, Campus de Teatinos s/n, 29071 Malaga, Spain; sergioml@uma.es (S.M.-L.); nextremera@uma.es (N.E.); 3 Department of Personality, Evaluation and Psychological Treatment, University of Malaga, Campus de Teatinos s/n, 29071 Malaga, Spain; lrey@uma.es; 4 Department of Psychology, University of Porto, Rua Alfredo Allen, s/n, 4200-135 Porto, Portugal; fneto@fpce.up.pt

**Keywords:** emotion regulation strategies, emotional intelligence, suicide risk, adolescence

## Abstract

Though contemporary scientific literature addressing the links between emotional intelligence (EI) and suicidal ideation in adolescents is scarce, one of the potential proposed pathways through which EI may reduce the risk of suicidal ideation involves its relationship with the use of adaptive coping strategies. The aim of this research is to provide support for an empirical pathway that proposes that the effects of EI on suicide risk may follow an indirect pathway, involving maladaptive and adaptive cognitive emotion regulation strategies, using both cross-sectional and prospective design in two independent studies with Spanish adolescents. The sample of Study 1 consisted of 1824 students (52.4% female; mean age 14.55 years). In Study 2, 796 adolescents (54.4% female; mean age 13.76 years) filled out the measures twice, four months later. The results confirmed a positive association between EI and adaptive cognitive emotion regulation strategies and a negative link with suicidal ideation. As expected, the results showed that both cross-sectionally (Study 1) and prospectively (Study 2) EI predicted lower suicidal ideation. Bootstrap mediation analysis indicated that only adaptive cognitive emotion regulation strategies partially mediated the link between EI and suicidal ideation both cross-sectionally and prospectively. Together, those adolescents who showed higher EI were more likely to report more adaptive cognitive emotion regulation, which in turn predicted lower levels of suicidal ideation. Our findings suggest possible avenues for prevention and intervention efforts aimed at boosting emotional abilities and developing adaptive coping strategies among adolescents who are at elevated suicide risk.

## 1. Introduction

Suicide is the second highest external cause of death among adolescents and young adults aged 15–29 years worldwide [1]. Previous meta-analytical research indicates that adolescence is associated with a greater incidence of suicidal ideation and suicide risk, indicating that suicidal ideation, suicide attempts and self-harm behaviours are strong predictors of death by suicide in adolescence, increasing the risk by approximately tenfold [2]. Thus, greater understanding of protective factors that could prevent the progression of suicide phenomena for developing optimal clinical intervention strategies during adolescence is required.

One especially important dimension that usually triggers feelings and thoughts related to suicide is the difficulty of expressing and regulating one’s own emotions [3,4,5]. Accordingly, emotional intelligence (EI), defined as a set of hierarchically organized emotional skills for perceiving, using, understanding and regulating emotions [6], has been shown to play a critical role in the prevention of suicidal behaviour/ideation during adolescence [7,8,9].

### 1.1. EI and Suicide

Accumulated evidence has shown consistent links between EI and the development and use of effective cognitive, affective and behavioural strategies that help individuals to cope with stress and negative events, leading to higher psychological health and well-being [10,11] and lower levels of suicidal ideation and suicide attempts [7,12,13]. During adolescence, EI has proved to be a protective factor for both suicidal ideation and suicide attempts in samples of adolescents without a history of traumatic events [8,14]. More interestingly, EI also has been found to mitigate the negative influence of cyberbullying and bullying on emotional adjustment (e.g., depressive symptomatology) [9,15] and suicidal ideation/behaviours [16]. Similarly, previous research confirmed that, among depressed adolescents, high EI protects against suicidal ideation [3]. In addition, Bonet et al. [7] found a significant decrease in suicidal ideation and hopelessness after applying an intervention programme focused on EI using a sample of adolescents in residential care. Taken together, these past findings suggest that emotional skills should make adolescents more confident that they can respond successfully to major and daily stressful events across different life areas (e.g., school, family, friendship, appearance), resulting in less suicidal ideation, fewer suicide attempts and higher psychological adjustment.

In line with the empirical evidence that emotionally intelligent adolescents report higher psychological health and lower suicidal ideation, it seems important to address the pathways by which EI operates. Previous studies have pointed out different potential mechanisms, including positive and negative affect [17,18] and coping strategies [13,19].

### 1.2. Cognitive Emotion Regulation Strategies, EI and Mental Health

Regarding the coping process, cognitive emotion regulation can be viewed as the cognitive part of coping, which involves the management of emotional information [20,21]. Garnefski et al. [20] conceptually introduced nine cognitive emotion regulation strategies divided into two further general categories: adaptive (i.e., acceptance, positive refocusing, focus on planning, putting into perspective and positive reappraisal) and maladaptive (i.e., blaming others, self-blame, catastrophizing and rumination) cognitive coping. Based on this approach, in adolescent samples, maladaptive strategies typically show positive links with mental and physical health problems, such as depressive symptomatology, anxiety and somatic complaints, whereas adaptive strategies positively relate to emotional well-being and better mental health indicators [21,22,23,24]. Furthermore, some of these studies also found a mediating role of maladaptive and/or adaptive cognitive emotion regulation strategies in the association between stressful or traumatic experiences and depressive symptomatology [25] and suicidal behaviour [24].

Previous empirical research suggests that EI may influence psychological health by providing the ability to overcome life stressors more successfully [3,13]. Therefore, individuals with high EI might exert a positive effect on suicidal ideation by using more effective and active coping strategies or engaging in lower maladaptive strategies than individuals with lower EI [26], which would impact positively on mental health outcomes [19,27]. For instance, Extremera et al. [19] have found recently that some adaptive cognitive emotion regulation strategies act as mediators in the associations between EI and well-being. At the same time, accumulating evidence suggests that the use of maladaptive coping strategies, the limited access to effective emotion regulation strategies and difficulties in regulating emotions are related to an increased risk of suicidal ideation [28,29,30].

Given the aforementioned connections, it is reasonable that adolescents with high EI levels would decrease their vulnerability towards suicidal ideation by using more adaptive cognitive strategies and lower maladaptive strategies. However, to date, no studies have examined the role of EI and emotion regulation cognitive strategies in explaining suicidal ideation in adolescence. In addition, there are still many gaps limiting our understanding of the underlying processes involved in EI and suicide in adolescence, given that much of the prior work has used adult samples and cross-sectional designs [8]. Thus, gaining knowledge about how EI and adaptive/maladaptive emotion regulation strategies are related to suicidal ideation among adolescents using prospective designs might be useful in order to measure and develop more tailored and effective programmes to prevent or minimize thoughts of death or suicide in future episodes. The present study aims to address these knowledge gaps.

### 1.3. This Study

**Figure 1:**
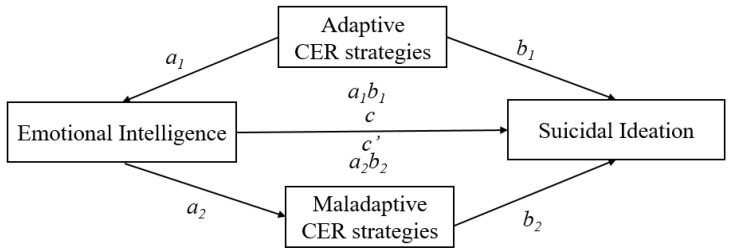
Illustration of the parallel multiple mediation model (Model 4; Hayes, 2018). *Note.* CER = cognitive emotion regulation.

Given the effect of EI on suicidal ideation, it is essential to understand the underlying causal mechanisms and processes in the relationship between EI and suicidal ideation in adolescents. Therefore, the main aim of this study was to provide additional empirical support for the potential underlying mechanisms in the relationship between EI and suicidal ideation, testing the role of cognitive coping strategies as mediators in two independent studies (Figure 1).

In Study 1, we examined, using cross-sectional design, whether adolescents’ cognitive emotion regulation strategies mediated the relationship between EI and suicidal ideation.

Study 2 was focused on whether EI was prospectively, in a 4-month period, associated with suicidal ideation, and if this link was mediated by adaptive and/or maladaptive cognitive emotion regulation strategies.

More specifically, according to the aforementioned theoretical and empirical findings, we hypothesized:

**Hypothesis** **1:** *EI would be positively correlated with adaptive cognitive emotion regulation strategies and negatively correlated with maladaptive cognitive emotion regulation strategies and suicidal ideation*.

**Hypothesis** **2:** *Cognitive emotion regulation strategies (both adaptive and maladaptive) would cross-sectionally mediate the relationship between EI and suicidal ideation (Study 1; n = 1824): that is, adolescents with high EI would tend to use more adaptive strategies and fewer maladaptive strategies, and in turn would engage in lower suicidal ideation*.

**Hypothesis** **3:** *Cognitive emotion regulation strategies would mediate the relationship between EI at Time 1 (T1) and suicidal ideation at Time 2 (T2) over time (Study 2; n = 796): that is, adolescents with high EI at T1 would tend to use more adaptive strategies and fewer maladaptive strategies at T2 and, in turn, be less prone to report suicidal ideation four months later*.

## 2. Materials and Methods

Data for the studies are based on two independent samples of adolescents with no overlap recruited from different high schools’ centers from South Spain. The sample of cross-sectional Study 1 comprised 1824 students (52.4% female; mean age 14.55 years) who completed the measures during December 2017 and March 2018 in nine high schools. In prospective Study 2, 796 adolescents (54.4% female; mean age 13.76 years) participated in two waves of data collection four months apart, which questionnaires were distributed in January 2019 (Time 1) and May 2019 (Time 2) in five high schools. Additional information about participants, measures, and procedure is provided in each Study.

## 3. Study 1

### 3.1. Participants

Sample was composed of 1824 students (52.4% female), ranging in age from 12 to 17 years (*M* = 14.55, *SD* = 1.67), from nine high schools in the autonomous community of Andalusia (Spain). The majority of the adolescents (93.5%) were born in Spain. Regarding academic grade, their distribution in compulsory secondary education (grades 1–4), high school (grades 5 and 6) and professional training was as follows: 17.4% in grade 1, 19.6% in grade 2, 17.1% in grade 3, 16.9% in grade 4, 17.8% in grade 5, 10.3% in grade 6 and 0.9% in professional training.

### 3.2. Measures

#### 3.2.1. Emotional Intelligence

EI was evaluated using the ‘Wong and Law Emotional Intelligence Scale’ (WLEIS [31]; Spanish validation by Extremera et al. [32]). The WLEIS is composed of 16 Likert-type items that have to be scored in the range 1–7 (i.e., from ‘totally disagree’ to ‘totally agree’). The scale comprises four subdimensions: self-emotional appraisal (SEA), other emotional appraisal (OEA), use of emotion (UOE) and regulation of emotion (ROE). Due to our interest in the overall EI construct, ratings on these four dimensions were summed to give the total score for this study. Cronbach’s α was 0.86 for the overall scale.

#### 3.2.2. Cognitive Emotion Regulation Strategies

Cognitive emotion regulation strategies were assessed using the ‘Cognitive Emotion Regulation Questionnaire’ (CERQ [20]; Spanish validation by Chamizo-Nieto et al. [33]). This questionnaire comprises 36 items and uses a five-point Likert scale (from 1, ‘almost never’, to 5, ‘almost always’) to evaluate two factors: adaptive and maladaptive emotion regulation strategies. Generally, the adaptive subscale covers five strategies: positive refocusing, putting into perspective, acceptance, refocus on planning and positive reappraisal. The maladaptive subscale covers four strategies: self-blame, blaming others, rumination and catastrophizing. For both the adaptive and maladaptive subscales, a high score indicates frequent use of the given strategies. For the present study, the subscales were categorized into two factors: adaptive and maladaptive strategies (adaptive CERQ and maladaptive CERQ, respectively). Both the adaptive CERQ (Cronbach’s α = 0.81) and maladaptive CERQ (Cronbach’s α = 0.74) demonstrated good internal consistency.

#### 3.2.3. Suicidal Ideation

Suicidal ideation was evaluated using the ‘Frequency of Suicidal Ideation Inventory’ (FSII [34]; Spanish validation by Sánchez-Álvarez et al. [35]). The FSII is composed of five Likert-type items that have to be scored in the range 1–5 (i.e., from ‘never’ to ‘almost every day’). This scale assesses how frequently individuals have considered suicidal thoughts over the previous 12 months. High scores indicate a high occurrence of suicidal ideation. In this study, Cronbach’s α was 0.88.

### 3.3. Procedure

This research was part of a larger project. Ethical approval and permission were obtained from the Research Ethics Committee of the host university before the start of the study (62-2016-H), in accordance with the Declaration of Helsinki [36]. To recruit the sample, secondary school management teams in the autonomous community of Andalusia were contacted and asked to collaborate. After their acceptance and approval, previous family consent was required for voluntary participation in this study. There was no family refusal for any adolescent’s collaboration. The pencil-and-paper questionnaires were completed in the school classrooms during the second and third trimester of the 2018 academic year in the presence of a teacher. Data collection was carried out by a member of the research team. At the beginning of the session, adolescents were informed about anonymity and confidentiality, as well as the instructions and the possibility of withdrawing from the study at any time.

### 3.4. Analysis

The data were analysed using the SPSS 24 statistical package. Descriptive statistics and Pearson’s correlation analysis are displayed in Table 1. Parallel multiple mediation analyses were conducted using Model 4 from the SPSS macro PROCESS [37] (see Figure 1). Following standard procedures [37], the indirect effect (path *ab*) was subjected to bootstrap analyses, producing bias-corrected 95% confidence intervals (CIs) of the effects from 10,000 resamples of the data to obtain more robust results due to lack of normality on the suicidal ideation scale. If the 95% CI of the indirect effect did not cover zero, the mediating effect was statistically significant at α = 0.05. In all analyses, we controlled for age, gender and academic grade. Unstandardized regression coefficients are reported.

### 3.5. Results

#### 3.5.1. Primary Analyses

Descriptive statistics (mean, standard deviation), Cronbach’s α and bivariate correlations among the study variables are presented in Table 1.

Regarding Hypothesis 1, the results show that EI was negatively related to suicidal ideation and positively associated with adaptive cognitive emotion regulation strategies. Suicidal ideation was negatively associated with adaptive cognitive emotion regulation strategies and positively correlated with maladaptive strategies. Adaptive strategies and maladaptive strategies showed a significantly positive relation to each other. However, maladaptive strategies were not significantly correlated with EI. Thus, Hypothesis 1 was partially supported.

#### 3.5.2. Mediation Analyses

Parallel multiple mediation analyses by bootstrapping were conducted to test Hypothesis 2, that adaptive and maladaptive strategies mediate the association between EI and suicidal ideation. Age, gender and academic grade were included as control variables.

As shown in Table 2, the mediation analyses showed significant indirect effects of EI on suicidal ideation via adaptive cognitive emotion regulation. Specifically, the results revealed that the coefficients of paths *a1* and *b1* were significant, indicating a positive association of EI on adaptive cognitive emotion regulation (*b* = 0.26, *p* < 0.001) and a negative relation between adaptive strategies and suicidal ideation (*b* = −0.13, *p* < 0.001). Moreover, significant indirect effects occurred for EI on suicidal ideation through adaptive cognitive emotion regulation strategies (path *a_1_b_1_*), with a point estimate of −0.03 (BCa 95% CI = −0.05 to −0.02). No significant indirect effects of maladaptive strategies (path *a_2_b_2_*) were found for the association between EI and suicidal ideation.

This cross-sectional mediation model accounted for 17% of the variance in suicidal ideation total scores. Therefore, adaptive CER strategies mediate the association between EI and suicidal ideation, and Hypothesis 2 is thereby partially supported.

## 4. Study 2

### 4.1. Participants

A sample of 1159 Spanish high school students (54.4% female), aged 12–17 years (*M* = 13.76, *SD* = 1.31) was recruited from five secondary schools in the Andalusian community. A total of 363 adolescents who participated in T1 did not participate in T2 (attrition rate = 31.32%). The final sample consisted of 796 students (55.5% female; *M* = 13.73, *SD* = 1.28). The majority of the adolescents (98%) were born in Spain. Regarding academic courses, their distribution by grade in compulsory secondary education (grades 1–4) was as follows: 26.8% in grade 1, 27.3% in grade 2, 23.1% in grade 3 and 22.9% in grade 4.

### 4.2. Measures

#### 4.2.1. Emotional Intelligence

EI was evaluated using the Spanish version of the WLEIS (see description in Study 1). Cronbach’s α was 0.88 (T1) for the overall scale.

#### 4.2.2. Cognitive Emotion Regulation Strategies

Cognitive emotion regulation strategies were assessed using the CERQ (see description in Study 1). The adaptive CERQ (Cronbach’s α: 0.88 for T1; 0.91 for T2) and maladaptive CERQ (Cronbach’s α: 0.80 for T1; 0.82 for T2) demonstrated good internal consistency.

#### 4.2.3. Suicidal Ideation

Suicidal ideation was evaluated using the FSII (see description in Study 1). Cronbach’s α was 0.92 (T2).

### 4.3. Procedure

Ethical approval and permission were obtained from the Research Ethics Committee of the host university prior to data collection (62-2016-H), in accordance with the Declaration of Helsinki [36]. To recruit the sample, the schools were selected by opportunity sampling based on their agreement to participate in this prospective study. Family consent was asked and given before trained graduate psychology (master’s and doctoral) students conducted the survey. The participants were guaranteed that their answers were anonymous and confidential, and they received instructions on the questionnaire, pointing out the possibility of withdrawing from the study at any time. The questionnaires were administered on paper and a code was assigned to each participant to enable linking of the T1 and T2 data. The second data collection point (T2) took place three to four months after the first, under the same conditions.

### 4.4. Analysis

The same data analyses for Study 1 were used for Study 2. The data were analysed using the SPSS 24 statistical package. Descriptive statistics and Pearson’s correlation analysis are displayed in Table 3. Parallel multiple mediation analyses were conducted using Model 4 from the SPSS macro PROCESS [37] (see Figure 1). As in Study 1, age, gender and academic grade were controlled and entered into the models as covariates. Unstandardized regression coefficients are reported.

### 4.5. Results

#### 4.5.1. Primary Analyses

The descriptive statistics (mean, standard deviation), Cronbach’s α and bivariate correlations between all the variables are presented in Table 3.

Regarding Hypothesis 1, the results indicate that EI was negatively related to suicidal ideation and positively associated with adaptive cognitive emotion regulation strategies (in both T1 and T2). Suicidal ideation was negatively associated with adaptive cognitive emotion regulation strategies (in both T1 and T2) and positively correlated with maladaptive cognitive emotion regulation strategies (T1) and maladaptive strategies (T2). Maladaptive cognitive emotion regulation strategies (T1 and T2) were not significantly associated with EI. Thus, Hypothesis 1 was partially supported.

#### 4.5.2. Mediation Analyses

Parallel multiple mediation analyses by bootstrapping were conducted to investigate Hypothesis 3. Each model included age, gender and academic grade as control variables.

Hypothesis 3 predicted that adaptive cognitive emotion regulation strategies (T2) and maladaptive strategies (T2) would mediate the association between EI (T1) and suicidal ideation (T2).

As shown in Table 4, the results revealed significant indirect effects of EI on suicidal ideation via adaptive cognitive emotion regulation strategies (T2). The coefficients of paths *a_1_* and *b_1_* were significant, indicating positive associations of EI on adaptive cognitive emotion regulation strategies (T2) (*b* = 0.21, *p* < 0.001) and of adaptive strategies (T2) on suicidal ideation (*b* = −0.18, *p* < 0.001), respectively. The total effect (path *c*) of EI on suicidal ideation was significant (*b* = −0.21, *p* < 0.001). The direct effect (path *c′*) remained significant (*b* = −0.16, *p* < 0.001) and the indirect effect (path *a_1_b_1_*) did not contain zero (*b* = −0.04, BCa 95% CI = −0.06 to −0.02), showing that adaptive cognitive emotion regulation strategies (T2) partially mediated the relationship between EI and suicidal ideation. No significant indirect effects of maladaptive cognitive emotion regulation strategies (path *a_2_b_2_*) were found for the association between EI and suicidal ideation. The final model accounted for 0.19% of the variance in suicidal ideation total scores (T2). Thus, Hypothesis 3 was supported.

In post-hoc analyses, we examined if adaptive cognitive emotion regulation strategies and maladaptive strategies would mediate the association between EI (T1) and suicidal ideation (T2) using the CERQ scores in T1. Results corroborated significant indirect effects of EI on suicidal ideation only via adaptive cognitive emotion regulation strategies (T1), with a point estimate of −0.07 (BCa 95% CI = −0.10 to −0.03). No significant indirect effects of maladaptive cognitive emotion regulation strategies (path *a_2_b_2_*) were found for the association between EI and suicidal ideation.

Taking the two studies together, adaptive coping strategies but not maladaptive coping strategies in T1 mediate the link between EI and suicidal ideation. These results replicated the same empirical pattern when the CERQ was used equally for both T1 and T2 as mediator in the link between EI (T1) and suicidal ideation (T2).

**Figure 2:**
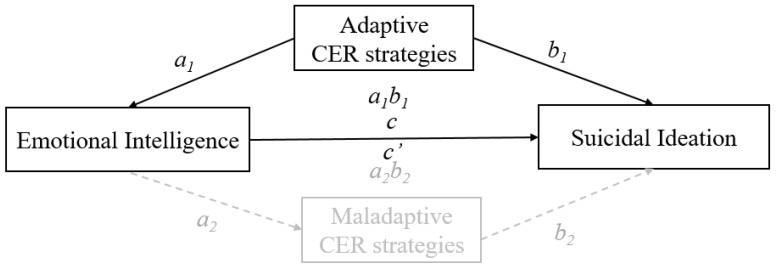
Illustration of the final mediation model: The mediating role of adaptive cognitive emotion regulation strategies in the relationship between EI and suicidal ideation.

In summary, the results of Studies 1 and 2 provide support for the mediational effect of adaptive cognitive emotion regulation strategies on the relationship between EI and suicidal ideation but not for the mediational effect of maladaptive cognitive emotion regulation strategies (Figure 2).

## 5. Discussion

The current study extended previous studies by examining the role of adaptive and maladaptive cognitive emotion regulation strategies in the relationship between EI and suicidal ideation in two samples of adolescents using cross-sectional (*n* = 1824) and prospective (*n* = 796) designs. Our research addresses important gaps within the research on EI and suicidal ideation, suggesting that adolescents with high EI are likely to develop more adaptive strategies that, in turn, contribute to decrease suicidal ideation.

Regarding Hypothesis 1, a positive link between EI and adaptive cognitive emotion regulation strategies was found. The results of Studies 1 and 2 support the notion that those adolescents who score higher on EI usually present more effective coping strategies (e.g., positive reappraisal, etc.) in response to stressors than their counterparts, who exhibit a lower confidence in their emotional abilities. These findings expand on and support past studies [19,27] indicating that people who are good at expressing and discriminating their emotions tend to cope more effectively in stressful situations of daily life, using more adaptive strategies for modifying the emotional state if desired. Furthermore, EI was negatively related to suicidal ideation in both studies. These findings are consistent with previous research suggesting that EI abilities seem to serve as protective factors against mental health problems and suicidal ideation [3,9,15].

With regard to cognitive emotion regulation strategies, maladaptive strategies were positively associated with suicidal ideation. Accordingly, previous studies have found negative associations between maladaptive strategies (e.g., rumination, self-blaming, etc.) and depressive symptomatology and suicide risk [29]. In contrast, adaptive strategies were negatively associated with suicidal ideation. This aligns with other past research [38] suggesting a positive and significant relationship between adaptive cognitive emotion regulation strategies and indicators of mental health and well-being. Taking the two studies together, our results provide preliminary support for the assumption of the protective role of EI and adaptive cognitive emotion regulation strategies on the emergence and progression of suicidal ideation [4,29].

According to Hypotheses 2 and 3, adaptive cognitive emotion regulation strategies showed a mediating role in the cross-sectional and prospective relationship between EI and suicidal ideation. However, no mediating role of maladaptive strategies was found. These results suggest that EI is associated with suicidal ideation only by means of its relationship with the use of adaptive cognitive emotion regulation strategies. Consistent with previous research, it may be that EI would influence mental health indicators by providing more effective ways to deal with life stressors [27]. Based on Fredrickson’s broaden-and-build theory, the ability to ‘intelligently’ use positive emotions and adaptive coping could serve as a means of guiding and understanding one’s behaviour and experience, reducing negative mood states and decreasing the frequency and intensity of suicidal thoughts, as well as improving well-being over time [39].

Consistent with our findings, when experiencing suicidal thoughts, people with high EI might be more prone to conceive of and enact a greater and more varied repertoire of integrative and positive thought patterns to cope more efficiently with suicidal thoughts and attempts [40]. These theoretical assertations are in line with empirical research conducted by Davis and Humphrey [27], who found that those individuals who feel more capable to use, understand and regulate their own and other’s emotions may develop more appropriate resources and are more able to effectively use strategies when modulating emotions to maintain better mental health. Therefore, for emotionally intelligent adolescents, it is plausible that EI may decrease or prevent the emergence of thoughts of death by: focusing on pleasant and joyful matter instead of a negative event (i.e., positive refocusing); establishing positive meaning to everyday situations in terms of personal growth (i.e., positive reappraisal); accepting what has happened and learning to live with the experience (i.e., acceptance); applying a strategy to handle future events (i.e., refocus on planning); and thinking and emphasizing the relativity and seriousness of the event (i.e., putting into perspective).

Despite past research linking EI with maladaptive strategies [21] and psychological problems with maladaptive strategies [4,22], in our two studies maladaptive strategies were not found to be significant mediators of suicidal ideation. Possible explanation may be related to the use of the broad concept of maladaptive strategies that did not distinguish specific ways of regulating emotions. As suggested by Compas et al. [4], future studies should replicate our results by providing more detailed descriptions of specific emotion regulation strategies that can be used by adolescents in order to extend knowledge about which of a given set of strategies may have the strongest effects on mental health. Alternatively, it is tentative to think that when both adaptive and maladaptive coping strategies are included in a multiple mediators model, the stronger component (adaptive coping strategies) acts as a mediator of the EI-suicidal ideation link (with the effect of maladaptive coping not being significant), suggesting that when both types of strategies are included in the model, adaptive coping strategies are most strongly associated with the appearance of suicidal ideation. Further studies should examine if positive and negative coping strategies, when linked to EI as mediators, might play independent and distinct roles in explaining suicidal ideation. It is also possible that the relationship between EI and suicidal ideation may be better explained by specific maladaptive strategies not included in the CERQ, such as behavioural emotion regulation (e.g., withdrawal, alcohol use, etc.) [41]. Future research should aim to replicate our findings using different maladaptive and adaptive behavioural coping and should consider employing positive psychological outcomes (e.g., flourishing, happiness, etc.) to expand our understanding of the complicated relationship between EI and positive and negative coping styles in adolescents at risk of mental health problems.

Overall, these findings may contribute to the literature regarding future intervention strategies used for adolescents at suicide risk and underline that, in our two studies, emotionally intelligent adolescents more frequently reported using adaptive coping strategies as a persisting protective resource, resulting in a considerable positive impact on frequency and intensity of suicidal ideation [27,42].

These results provide relevant implications for the current understanding of the underlying processes involved in the EI-suicide link in adolescence and thus suggest future avenues for preventing suicide. First, identifying adolescents who lack the emotional ability to deal with daily stressors would be useful for school counsellors to potentially reduce the risk of suicidal ideation. For example, counsellors should use screening tests or structured interviews for the absence of protective factors such as EI and adaptive cognitive strategies, together with the routine assessment of social, academic or familial problems. Second, according to our findings, it may be beneficial for interventions to focus on enhancing emotional abilities and adaptive coping strategies rather than just eliminating the maladaptive coping strategies. Given that prior empirical evidence suggests the need for training on socio-emotional competency in schools to improve the well-being of specific at-risk groups [43], targeting the deficiencies in adaptive emotion regulation strategies may play an important role in EI interventions aiming to prevent suicidal ideation. The cognitive model of suicidal behaviour states that emotion and subsequent behaviour are influenced by the manner in which situations are interpreted [44]. Thus, the use of adaptive/maladaptive cognitive coping strategies might trigger these interpretations and contribute to the appearance/reduction of suicidal ideation. Therefore, school counsellors and therapists who work with adolescents might offer EI intervention programmes focusing on effective cognitive ways of handling major and daily stressful events during adolescence (e.g., through workshops), aimed at restructuring such dysfunctional cognitions in order to reduce suicidal thoughts.

### Limitations

Although the present study provides meaningful knowledge on the cross-sectional and prospective relationships between EI, cognitive emotion regulation strategies and suicidal ideation in adolescence, some limitations should be acknowledged. Firstly, although the main strength of this study was the use of two samples and two designs (i.e., cross-sectional and prospective) to examine the conceptual model, a longer term study design (e.g., 6, 12 or more months) would help to examine the differential effects of EI and specific cognitive emotion regulation strategies on suicidal ideation.

Secondly, both studies used self-reports and non-clinical samples of adolescents who participated voluntarily, thus it is possible that the self-perception of some adolescents might be biased. However, the self-report measures showed good reliability and the sample sizes are quite adequate and representative of the adolescent population in order to consider significant findings that could help researchers to improve their knowledge of the link between EI and suicidal ideation. The use of structured interviews or other informants (e.g., parents and teachers) will definitely benefit future understanding in the coping pattern involving EI and suicide. Moreover, it should be acknowledged that suicidal ideation is not the solely predictor of suicide, thus future studies might examine the links EI-cognitive emotion regulation on suicide attempts and behaviors [8,45]. It is also necessary to involve clinical or community samples of young people who may be at risk of committing suicide [3].

Finally, it would be useful to use longer follow-up randomly designed studies to examine specific cognitive emotion regulation strategies in the relationship between EI and suicidal ideation as they may vary in effectiveness for preventing suicidal ideation. In the same vein, future longitudinal research should be developed to identify and evaluate sociodemographic variables such as gender and age differences, as well as other relevant factors (i.e., contextual or individual) that seem to present causal effects on suicidal ideation and other important internalizing problems in adolescence (e.g., loneliness, anxiety, etc.).

## 6. Conclusions

The results of our study underline the importance of assessing complex relationships between emotional abilities affecting suicidal ideation among adolescents and point to the potential role of adaptive cognitive emotion regulation strategies in the relationship between EI and suicidal ideation. Although the results need replication and further analyses, this research expands our prior knowledge based on EI research in adolescents, highlighting that adaptive cognitive coping strategies may be important mechanisms for explaining why EI is positively related to better mental health indicators and reduced suicidal ideation. These findings address new directions and implications for intervention in the management and prevention of suicidal ideation during adolescence.

## Figures and Tables

**Table 1:** Descriptive statistics of the Study 1.

Variables	1	2	3	4	*M (SD)*	Range	α
1. Emotional Intelligence	-				4.82 (0.94)	1.19–7	0.86
2. Adaptive CER strategies	0.39 **	-			3.22 (0.61)	1.55–4.85	0.81
3. Maladaptive CER strategies	−0.02	0.23 **	-		2.69 (0.54)	1.13–4.56	0.74
4. Suicidal Ideation	−0.26 **	−0.12 **	0.27 **	-	1.65 (0.79)	1–5	0.88

*Note*. CER = cognitive emotion regulation. ** *p* < 0.01.

**Table 2:** Summary of the indirect effects between EI and suicidal ideation through CER strategies (Study 1).

Antecedent	Consequent														
		*M_1_* (Adaptive CER Strategies)					*M_2_* (Maladaptive CER Strategies)					*DV* (Suicidal Ideation)			
		B	SE	*t*	BCa 95% CI		B	SE	*t*	BCa 95% CI		B	SE	*t*	BCa 95% CI
*IV* (EI)	*a_1_*	0.26	0.01	18.13 ***	(0.23, 0.28)	*a_2_*	−0.01	0.01	−0.55	(−0.03, 0.02)	*c’*	−0.16	0.02	−8.24 ***	(−0.20, −0.13)
*M_1_* (Adaptive CER strategies)											*b_1_*	−0.13	0.03	−4.20 ***	(−0.19, −0.07)
*M_2_* (Maladaptive CER strategies)											*b_2_*	0.41	0.03	12.72 ***	(0.35, 0.47)
Constant		1.33	0.27	5.00 ***	(0.81, 1.85)		2.57	0.26	9.95 ***	(2.07, 3.08)		0.72	0.35	2.03 *	(0.02, 1.41)
Age ^a^		0.06	0.02	2.83 **	(0.02, 0.10)		0.01	0.02	0.53	(−0.03, 0.05)		0.06	0.03	2.05 *	(0.00, 0.11)
Gender ^a^		−0.03	0.03	−1.00	(−0.08, 0.03)		0.04	0.03	1.50	(−0.01, 0.09)		0.22	0.03	6.27 ***	(0.15, 0.28)
Grade ^a^		−0.05	0.02	−2.47 *	(−0.10, −0.01)		−0.02	0.02	−0.86	(−0.06, 0.02)		−0.03	0.03	−1.18	(−0.09, 0.02)
Total effect											*c*	−0.20	0.02	−10.57 ***	(−0.24, −0.16)
Indirect effect	*a_1_b_1_*	−0.03	0.01		(−0.05, −0.02)	*a_2_b_2_*	−0.00	0.01		(−0.02, 0.01)	*ab*	−0.04	0.01		(−0.06, −0.02)
R^2^		0.16					0.00					0.17			
F (*df*)		85.37 *** (4, 1819)					0.91 (4, 1819)					59.64 *** (6, 1817)			

*Notes. N* = 1824. Abbreviations *DV* = Dependent Variable; *IV* = Independent Variable; *M* = Mediator; *a*, *b*, *c*, and *c*’ represent unstandardized regression coefficients; CER = cognitive emotion regulation. ^a^ Control variables. BCa 95% CI = bias-corrected and accelerated 95% confidence interval. Bootstrap sample size = 10,000; * *p* < 0.05, ** *p* < 0.01, *** *p* < 0.001.

**Table 3:** Descriptive statistics of the Study 2.

Variables	1	2	3	4	5	6	*M* (*SD*)	Range	α
1. Emotional Intelligence (T1)	-						4.84 (1.02)	1.25–7	0.88
2. Adaptive CER strategies (T1)	0.47 **	-					3.38 (0.71)	1–5	0.88
3. Adaptive CER strategies (T2)	0.28 **	0.47 **	-				3.24 (0.79)	1–5	0.91
4. Maladaptive CER strategies (T1)	−0.02	0.34 **	0.14 **	-			2.67 (0.63)	1–5	0.80
5. Maladaptive CER strategies (T2)	−0.04	0.16 **	0.44 **	0.49 **	-		2.57 (0.63)	1–5	0.82
6. Suicidal Ideation (T2)	−0.23 **	−0.11 **	−0.03	0.30 **	0.32 **	-	1.64 (0.90)	1–5	0.92

*Note*. CER = cognitive emotion regulation; T1 = Time 1 (*N* = 1159); T2 = Time 2 (*N* = 796). * *p* < 0.05; ** *p* < 0.01.

**Table 4:** Summary of the indirect effects between EI (T1) and suicidal ideation (T2) through CER strategies (T2) (Study 2).

Antecedent	Consequent														
		*M_1_* (Adaptive CER Strategies T2)					*M_2_* (Maladaptive CER Strategies T2)					*DV* (Suicidal Ideation T2)			
		B	SE	*t*	BCa 95% CI		B	SE	*t*	BCa 95% CI		B	SE	*t*	BCa 95% CI
*IV* (EI)	*a_1_*	0.21	0.03	8.02 ***	(0.16, 0.26)	*a_2_*	−0.03	0.02	−1.34	(−0.07, 0.01)	*c’*	−0.16	0.03	−5.28 ***	(−0.22, −0.10)
*M_1_* (Adaptive CER strategies)											*b_1_*	−0.18	0.04	−4.08 ***	(−0.26, −0.09)
*M_2_* (Maladaptive CER strategies)											*b_2_*	0.52	0.05	9.90 ***	(0.42, 0.63)
Constant		2.93	0.50	5.92 ***	(1.96, 3.90)		2.21	0.41	5.40 ***	(1.41, 3.01)		0.69	0.55	1.24	(−0.40, 1.77)
Age ^a^		−0.05	0.03	−1.71	(−0.11, 0.01)		0.03	0.02	1.14	(−0.02, 0.08)		0.04	0.03	1.14	(−0.03, 0.10)
Gender ^a^		0.14	0.05	2.64 **	(0.04, 0.25)		0.12	0.04	2.61 **	(0.03, 0.20)		0.27	0.06	4.55 ***	(0.15, 0.38)
Grade ^a^		−0.05	0.02	−3.23 **	(−0.08, −0.02)		−0.02	0.01	−1.40	(−0.04, 0.01)		0.01	0.02	0.33	(−0.03, 0.04)
Total effect											*c*	−0.21	0.03	−7.02 ***	(−0.27, −0.15)
Indirect effect	*a_1_b_1_*	−0.04	0.01		(−0.06, −0.02)	*a_2_b_2_*	−0.02	0.01		(−0.04, 0.01)	*ab*	−0.05	0.02		(−0.09, −0.02)
R^2^		0.10					0.03					0.19			
F (*df*)		22.59 *** (4, 791)					5.24 *** (4, 791)					30.73 *** (6, 789)			

*Notes. N* = 796. Abbreviations *DV* = Dependent Variable; *IV* = Independent Variable; *M* = Mediator; *a*, *b*, *c*, and *c*’ represent unstandardized regression coefficients; CER = cognitive emotion regulation. ^a^ Control variables. BCa 95% CI = bias-corrected and accelerated 95% confidence interval. Bootstrap sample size = 10,000; ** *p* < 0.01, *** *p* < 0.001.
